# Evaluation of iStent Micro-Bypass vs. Kahook Dual Blade Goniotomy with Phacoemulsification in Open-Angle Glaucoma: A Systematic Review

**DOI:** 10.3390/jcm14165819

**Published:** 2025-08-18

**Authors:** Anna Charytonowicz, Jakub Błażowski, Joanna Konopińska

**Affiliations:** Department of Ophthalmology, Medical University of Bialystok, 15-276 Bialystok, Poland; nesterukanna@gmail.com (A.C.); blazowski.jakub@gmail.com (J.B.)

**Keywords:** intraocular pressure, iStent, Kahook Dual Blade, minimally invasive glaucoma surgery, open-angle glaucoma, phacoemulsification

## Abstract

**Background/Objectives**: Glaucoma refers to a group of eye diseases that damage the optic nerve, causing irreversible vision loss. It typically begins with peripheral vision impairment and, in severe cases, leads to complete blindness. A major advancement in glaucoma treatment is Microinvasive Glaucoma Surgery (MIGS), including trabecular bypass and ab interno trabeculectomy, which are generally used for mild to moderate glaucoma. This review aimed to evaluate the efficacy and safety of iStent micro-bypass implantation and Kahook Dual Blade (KDB) goniotomy combined with phacoemulsification in patients with open-angle glaucoma (OAG). **Methods**: A review of recent studies was conducted using PubMed, Google Scholar, Scopus, Web of Science, and Embase. Both prospective and retrospective clinical studies were included. These MIGS methods were compared for reducing intraocular pressure (IOP) and medication burden at baseline and endpoint. **Results**: Eleven studies involving 1925 eyes were analyzed. All studies showed that iStent (first- and second-generation) micro-bypass implantation and KDB goniotomy reduced IOP, favoring the phaco-KDB group. Antiglaucoma medication use also decreased significantly. The success rate was sufficient and most complications were minimal. **Conclusions**: In conclusion, iStent implantation and KDB goniotomy offer a high safety profile, meaningful IOP reduction, a minimally invasive approach, and quick recovery.

## 1. Introduction

Glaucoma is a group of eye diseases and the leading cause of irreversible blindness. It is characterized by gradual degeneration of the optic nerve and is often associated with elevated intraocular pressure (IOP) [1,2]. Open-angle glaucoma (OAG) is the most frequent type of glaucoma, and the first-line treatment involves medications to lower ocular hypertension. When medications fail or result in severe side effects, antiglaucoma surgery becomes the best alternative. Trabeculectomy has long been regarded as the gold standard among filtering surgical techniques. However, it is associated with significant risks and complications, such as endophthalmitis, choroidal hemorrhage [2], hypotony [3], bleb fibrosis, and the need for reoperation. Microinvasive Glaucoma Surgery (MIGS) refers to the surgical techniques that use an ab interno approach, minimize tissue disruption, ensure a favorable safety outcomes, and support rapid postoperative recovery [4]. The iStent micro-bypass implantation and KDB goniotomy are two MIGS techniques that aim to improve aqueous humor outflow through Schlemm’s canal (SC) and are often performed in combination with phacoemulsification [5].

The iStent is one of the earliest and most widely used MIGS devices for patients with OAG. Initially introduced in 2012, this micro-bypass began as a first-generation stent implanted singly. A few years later, Glaukos Corporation (Laguna Hills, CA, USA) developed the second-generation iStent inject, which incorporates two stents [5]. This small device, made of heparin-coated titanium, features a central lumen and four side holes (second-generation) or lumen and retention arches (first-generation) to enhance aqueous humor drainage through SC. It is implanted in the nasal part of the trabecular meshwork (TM) via an ab interno approach, improving the natural filtration system responsible for aqueous humor drainage. The device creates a direct passage in the TM, facilitating aqueous humor flow into SC and collector channels, thereby lowering IOP. The procedure is typically performed immediately following cataract surgery.

Kahook Dual Blade (KDB, New World Medical, Rancho Cucamonga, CA, USA) goniotomy is a technique that uses a surgical blade designed to simultaneously remove a part of the TM, creating a clear pathway for aqueous humor drainage into SC. An ab interno approach is also utilized in this method. The device’s blades are its key feature: one is designed to extract a portion of the TM, while the other pushes the tissue aside. The knife is engineered to minimize the risk of damaging adjacent tissues [6]. This procedure does not require an implant but instead removes the inner part of SC over a wide area (approximately at 100° of the SC circumference), potentially providing access to multiple collector channels. Consequently, ab interno goniotomy results in a reduction in IOP. The KDB goniotomy can also be performed in combination with phacoemulsification.

The aim of this review was to analyze the efficacy and safety of iStent micro-bypass implantation versus KDB goniotomy in OAG patients by reviewing recent studies. This review paper directly compares iStent micro-bypass (including first- and second-generation stents) with goniotomy using KDB as a combined procedure with phacoemulsification.

## 2. Materials and Methods

### 2.1. Search Procedure and Information Extraction

This review followed the PRISMA (Preferred Reporting Items for Systematic Review and Meta-Analysis) guidelines and was registered on the PROSPERO international prospective register of systematic reviews (PROSPERO registration number: CRD 1045634).

**Figure 1:**
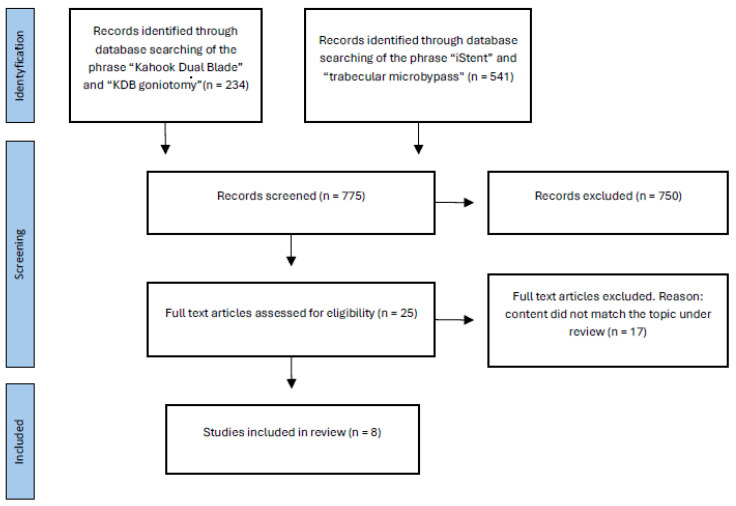
PRISMA flowchart of the study selection procedure.

The following databases were searched: PubMed, Google Scholar, Scopus, Web of Science, and Embase. The search terms used were “glaucoma,” “MIGS,” “open-angle glaucoma,” “primary open-angle glaucoma,” “iStent,” “trabecular microbypass,” “Kahook Dual Blade,” and “KDB goniotomy.” Prospective and retrospective publications were searched and independently evaluated by two authors (J.K. and A.C.). Reviews, meta-analyses, studies reporting partial results, and those omitting specific analyzed factors were excluded. Based on abstract analysis, full-length texts of the articles relevant to the topic were retrieved. Only articles in English were evaluated without restrictions on publication date. The specific number of data points at each search step is shown in Figure 1 (PRISMA flowchart). Participants included adults aged 18 years or more with a confirmed diagnosis of glaucoma (e.g., primary open-angle glaucoma, normal-tension glaucoma, secondary glaucoma), identified based on clinical or diagnostic criteria. Any discrepancies between reviewers were resolved by discussion.

The following data were collected from the included trials: the primary author, year of publication, patient age, patient population, baseline IOP, baseline antiglaucoma medications, endpoint IOP, endpoint antiglaucoma drops, iStent generation, the number of implants inserted, and follow-up duration. Primary outcome measures were percentage reduction in IOP and antiglaucoma medications at the end of follow-up. Secondary outcome measures were surgical success and the complication rate.

### 2.2. Inclusion Criteria

- Prospective or retrospective clinical studies.
- The studies involved patients with OAG.
- Used procedures: iStent micro-bypass implantation with phacoemulsification or KDB goniotomy with phacoemulsification.
- The trial compared the following criteria: baseline IOP, endpoint IOP, baseline number of glaucoma drops, number of endpoint glaucoma drops, and best-corrected visual acuity.
- At least 6 months minimum follow-up period.

### 2.3. Exclusion Criteria

- Meta-analyses and reviews.
- Studies describing partial results.
- Studies omitting certain analyzed factors.
- Case reports.

### 2.4. Outcome Measures

Since different authors applied diverse success criteria, we defined the target IOP (<18 mmHg) at the end of follow-up without medications as a complete success, and qualified success was set as the target IOP (<18 mmHg) at the end of follow-up with or without medications. For the safety assessment, the percentage of patients with postoperative adverse events were analyzed.

### 2.5. Quality Assessment

Studies were assessed independently by two authors with a consensus to resolve differences. The Cochrane Handbook for Systematic Reviews of Interventions (version 5.1.0) risk of bias tool was employed to assess the methodological quality of the studies. Each included study was evaluated for selection bias, performance bias, detection bias, attrition bias, reporting bias, and other biases. Additionally, we used GRADE assessments which incorporate risk of bias, directness of evidence, consistency and precision of results, and possibility of publication bias to evaluate the certainty of the evidence (Table 1).

## 3. Results

### 3.1. iStent Micro-Bypass Implantation vs. Kahook Dual Blade Goniotomy

From the available literature, we selected five studies [6,7,8,9,10] that directly compared iStent implantation (phaco-iStent) with goniotomy using KDB (phaco-KDB) (Table 2).

Barkander et al. [6] analyzed iStent inject implantation (second-generation) versus KDB goniotomy, both combined with phacoemulsification. This study evaluated a Caucasian patient population with primary open angle glaucoma (POAG), pseudoexfoliation glaucoma (PEXG), and pigmentary glaucoma (PG). The phaco-iStent group was smaller (n = 56) than the phaco-KDB group (n = 97). An IOP reduction of ≥20% with IOP ≤ 18 mmHg was achieved by 51% of the phaco-KDB group and 46% of the phaco-iStent group. After 24 months, both groups showed a significant decrease in medication use; however, a larger reduction of ≥1 medication was observed in the phaco-KDB group (53% vs. 32% in the phaco-iStent group). Additionally, a greater proportion of patients in the phaco-KDB group were medication-free at 2 years. Hyphema was the most common adverse event reported in this study (14% in the phaco-iStent group, 62% in the phaco-KDB group). Seven eyes presented large hyphema (>2 mm) with vision impairment (<20/200), and, interestingly, all occurred exclusively in eyes with PEXG. Other postoperative complications included IOP spikes, macular edema, anterior chamber flare, and additional glaucoma surgery. Among cases requiring secondary surgical intervention, only 27% involved PEXG, which was a surprising result given the typically greater aggressiveness of this glaucoma type.

One of the largest retrospective trials on this subject was conducted by Dorairaj et al. [7], who compared IOP outcomes in eyes operated with phacoemulsification combined with KDB goniotomy or first-generation iStent implantation. The study included 435 eyes diagnosed with cataract and glaucoma, which were divided into two groups: phaco-KDB (n = 237) and phaco-iStent (n = 198). The study showed an average reduction of 5.0 mmHg in mean IOP in the phaco-KDB group (from 17.9 ± 4.4 mmHg at baseline to 13.6 ± 2.7 mmHg after 12 months) and an average reduction of 2.3 mmHg in the phaco-iStent group (from 16.7 ± 4.4 mmHg at the pre-surgery to 13.9 ± 2.7 mmHg at the end of follow-up). The average number of glaucoma medications also changed, with the phaco-KDB group representing an average decrease of 1.1 medications, which corresponded to a 63% reduction, and with the phaco–iStent group representing an average decrease of 0.9 medications, which equaled a 46% reduction, respectively. Phaco-KDB eyes experienced an approximately 50% greater IOP reduction and a 35% larger decrease in glaucoma medications compared to phaco-iStent eyes. The most common postoperative complication was IOP elevation, observed in 12.6% of the phaco-iStent group and 6.3% of the phaco-KDB group. Other adverse events included corneal edema, posterior capsular opacification (PCO), anterior chamber inflammation, and posterior vitreous detachment.

El Mallah et al. [8] examined Caucasian patients undergoing cataract surgery with KDB goniotomy versus first-generation iStent trabecular bypass implantation. This retrospective analysis included eyes with mild to moderate glaucoma: 190 in the phaco-KDB group and 125 in the phaco-iStent group. Clinically meaningful reductions in mean IOP and glaucoma medications were observed consistently at all time points in both groups. By the end of the follow-up, mean IOP reduction was more pronounced in the phaco-KDB group (27%) compared to the phaco-iStent group (13.7%). A key feature of this study was subgrouping by baseline IOP: ≤18 mmHg and >18 mmHg. Subgroup analysis revealed a significant medication reduction in the lower IOP group and reductions in both IOP and medications in the higher IOP group. A ≥1 medication reduction was achieved by 80.4% of phaco-KDB eyes and 77.4% of phaco-iStent eyes. The most common side effects were transient anterior chamber flare and IOP elevation, both resolving without additional surgery.

Lee et al. [9] conducted a retrospective study of 44 phaco-KDB eyes and 58 phaco-iStent eyes, most with mild POAG. After 6 months, 43.2% of the phaco-KDB group versus 17.2% of the phaco-iStent group achieved surgical success, defined as a reduction of ≥1 medication while maintaining IOP ≤ 18 mmHg. This study also highlights the high safety profile of these two MIGS methods. The most common complication was elevated IOP (18.2% in phaco-KDB eyes), potentially related to steroid use. One hypothesis of this study suggests that cells forming distal outflow pathways, such as Schlemm’s canal, episcleral veins, and collector channels, may contract like blood vessels and significantly influence IOP regulation [17,18].

Another direct comparison of the phaco-KDB and phaco-iStent groups was performed by Iwasaki et al. [10] in a retrospective trial of POAG and PXG eyes. This analysis used criterion A (<20% IOP reduction from baseline or IOP > 18 mmHg) and criterion B (<20% IOP reduction from baseline or IOP > 14 mmHg). The criterion A success rate after 12 months was higher in the phaco-KDB group (60.2%) than that in the phaco-iStent group (46.4%). However, no notable differences were observed for criterion B. Medication reduction was greater in the phaco-iStent group, likely owing to the phaco-KDB group having more severe glaucoma cases requiring additional medications to sustain target IOP. Predominant postoperative complications included hyphema and IOP spikes, which were significantly more frequent in the phaco-KDB group.

### 3.2. iStent Micro-Bypass Implantation

Gaskin et al. [11] compared cataract surgery (control group, n = 48) with iStent inject insertion combined with phacoemulsification (study group, n = 56) in a prospective, randomized trial with 24 months follow-up. Similar favorable outcomes regarding IOP reduction were observed. In combined surgery cases, IOP decreased to 14.6 ± 3.7 mmHg (from 17.1 ± 3.1 mmHg at baseline) 2 years post-surgery, while in the control group, IOP decreased to 15.2 ± 5.8 mmHg (from 17.7 ± 4.0 mmHg at baseline). Although IOP declined more after 4 weeks in the study group, there was no considerable difference between groups after 24 months. This demonstrates that cataract surgery alone can also reduce IOP. In the study group, fewer glaucoma medications were required on average; moreover, 57% of eyes in the study group were medication-free compared to 36% of eyes in the control group that were medication-free. The main postoperative complications included secondary interventionssuch as anterior chamber irrigation owing to retained cortical masses, selective laser trabeculoplasty, trabeculectomy, and IOL exchange, and there were no differences in the complication rate between the groups. No cases of postoperative stent displacement were reported; however, intraoperatively, three stents had to be implanted in one eye owing to injector malfunction and inadequate positioning of the first stent. In another eye, a single stent was placed after one was displaced and removed during surgery. Furthermore, the coronavirus disease pandemic reduced the assumed trial size owing to recruitment challenges and social distancing restrictions.

A comprehensive, retrospective multicenter study by Clement et al. [12] evaluated the safety and efficacy of iStent micro-bypass insertion with phacoemulsification in the Australian population and demonstrated positive results in IOP and medication reduction. The authors analyzed eyes with cataract; mild to advanced glaucoma: POAG, primary angle closure (PAC), or normal-tension (NTG) glaucoma; and ocular hypertension for 36 months of follow-up. Among 273 treated eyes, the average IOP decreased by 15.5%, and antiglaucoma medication use dropped by 68.5%. Over 70% of eyes achieved an IOP of ≤15 mmHg (compared to 49.1% pre-surgery), and 70% of eyes were medication-free (compared to 21.6% pre-surgery). Subgroup analysis showed satisfactory effectiveness across glaucoma types, with IOP reduction ranging from 13 to 22% and medication reduction from 42 to 94%. Most adverse events occurred early postoperatively, with only three events reported after the first month. Over 36 months of follow-up, 20 eyes underwent secondary antiglaucoma surgery: 12 eyes SLT (4.4%), 5 eyes (1.8%) trabeculectomy or tube insertion, and 3 eyes (1.1%) required both SLT and a filtering surgery.

Another notable prospective, randomized, two-center study by Kozera et al. [13] compared iStent implantation combined with phacoemulsification (study group) to phacoemulsification as a standalone procedure (control group). The division into two study subgroups was based on preoperative IOP: one group with <26 mmHg and another group with ≥26 mmHg. After 24 months, a larger drop in IOP was observed in the study group, and a similar effect was noted for the reduction in antiglaucoma medication. In all participants, the mean IOP dropped by 6 mmHg (27%): from 22.05 ± 2.40 mmHg at baseline to 16.20 ± 2.37 mmHg on average after 24 months of follow-up. In patients in the IOP < 26 mmHg subgroup, the average IOP declined by 5.0 mmHg (25.8%): from 21.03 ± 1.44 mmHg at baseline to 15.60 ± 2.12 mmHg at the end of follow-up. In patients in the IOP ≥ 26 mmHg subgroup, the average IOP declined by 7.00 mmHg (−28.6%): from 26.00 ± 0.00 mmHg at baseline to 18.56 ± 1.81 mmHg. The average postoperative IOP was higher in patients in the IOP ≥ 26 mmHg subgroup compared to those in the IOP < 26 mmHg subgroup, likely owing to SC collapse and reduced patency of distal outflow routes from the anterior chamber owing to high IOP. No notable intraoperative adverse events occurred after iStent implantation combined with cataract surgery. Postoperative complications included microhyphema, corneal edema, PCO, and age-related macular degeneration progression. This study concluded that patients with IOP in the low twenties (26 mmHg) will benefit most from iStent surgery, from whom both IOP levels and reduction in medication burden are the most advantageous. For patients with high baseline IOP (>26 mmHg), it may be appropriate to choose another type of surgery. To the best of our knowledge, this was the only study conducted with a pre-surgery wash out.

Each of the mentioned iStent studies documented a decrease in the average number of antiglaucoma medications used post-surgery compared to pre-surgery. A similar trend was observed in the postoperative reduction in IOP. Detailed data are presented in Table 3 and Table 4.

### 3.3. Kahook Dual Blade Goniotomy

A 2023 retrospective study by Barkander et al. [15] examined KDB goniotomy as a standalone procedure or in conjunction with phacoemulsification in 90 eyes with POAG or PEXG. The research showed a notable decrease in IOP after 24 months, from 24.8 ± 8.3 to 15.0 ± 5.3 mmHg in the KDB-solo eyes and from 22.3 ± 5.8 to 13.9 ± 3.0 mmHg in the phaco-KDB eyes. The amount of medication also declined from 3.5 ± 0.6 to 3.1 ± 0.9 in the KDB-solo group and from 3.3 ± 0.5 to 2.3 ± 1.1 in the phaco-KDB group. After the follow-up period, 5% of KDB-solo eyes were medication-free, compared to 14% in the phaco-KDB group. Eyes in the phaco-KDB group achieved twice the beneficial percentage as the KDB-alone group concerning the following criterion: a reduction in IOP of more than 20% and/or a reduction of ≥1 antiglaucoma eyedrops. At the end of follow-up, IOP levels and medication burden were similar in POAG and PEXG eyes. IOP spikes and hyphema were the most common complications but resolved spontaneously without requiring additional interventions. The hyphema mentioned above was associated with anticoagulation drugs in the KDB-solo group.

A study conducted by Iwasaki et al. [15] in 2022 investigated a larger group (n = 148) and provided a longer follow-up for the KDB goniotomy procedure combined with phacoemulsification in patients with POAG and PEXG. After 31.3 ± 14.8 months, the mean IOP decreased to 11.9 ± 2.7 mmHg. IOP was higher in PEXG eyes than in POAG eyes both before surgery and at 36 months postoperatively. A 33% reduction in glaucoma medication was reported after surgery, with a higher rate of medication-free eyes in the POAG group. The success rates for achieving IOP < 18 mmHg and <14 mmHg were 52.5% and 36.9%, respectively.

Dorairaj et al. [19] conducted a prospective study involving eyes with OAG from seven centers in North America. The patients underwent phacoemulsification combined with goniotomy using KDB. After 12 months, IOP was reduced from 16.8 ± 0.6 mmHg at baseline to 12.4 ± 0.3 mmHg, with a 50% reduction in glaucoma medications. The study divided patients into two subgroups: one with ≤16.5 mmHg and another with >16.5 mmHg baseline IOP. In the higher IOP group, the primary goal was to reduce IOP; however, in the lower IOP group, the primary goal was to reduce medication burden. Success was achieved in both subgroups: in the lower IOP group, 85% of eyes reduced medications by one or more, while in the higher IOP group, all eyes achieved a minimum IOP reduction of 20%. The most frequent side effects were pain/irritation, PCO, and IOP spikes > 10 mmHg. Cystoid macular edema and glare were reported much less frequently.

All the mentioned KDB goniotomy studies demonstrated a reduction in the number of antiglaucoma drops used after surgery. This is comparable to the postoperative reduction in IOP. Detailed data are presented in Table 5.

## 4. Discussion

Our review examined the effects of iStent insertion and KDB goniotomy on lowering IOP and reducing glaucoma medications based on 11 studies gathered through a database search.

Table 2, Table 3 and Table 4 present the features of the included studies. The follow-up ranged from 6 to 36 months, with a total of 1925 eyes analyzed. The first-generation iStent was implanted in 451 eyes, and second-generation in 385 eyes. The number of iStent bypasses used varied; 451 eyes received one stent, 381 eyes had two stents, and 1 eye received three stents. Among iStent studies, the highest baseline IOP was 22.04 ± 1.64 mmHg, and the lowest endpoint IOP was 13.9 ± 3.5 mmHg. For KDB goniotomy, the baseline IOP was 22.3 ± 5.8 mmHg, and the endpoint IOP was 11.9 ± 2.7 mmHg. The range of antiglaucoma drug usage was 3.0 ± 0.9 at baseline to 0.32 ± 0.55 at the endpoint in iStent studies and 3.3 ± 0.5 to 0.43 ± 0.05 in KDB goniotomy studies.

Studies showed a significant difference in IOP lowering and antiglaucoma medication reduction, favoring the phaco–KDB procedure. This greater effect may be due to goniotomy’s access to more collector channels than iStent implantation [6]. Additionally, earlier studies indicated that previously placed iStents may not always function effectively due to suboptimal positioning, which can influence IOP-lowering capability [19]. Moreover, the iStent valve could experience overgrowth; the situation is different in KDB goniotomy which is an implant-free procedure where there is no basis for foreign-body granuloma or tissue overgrowth around an implant. The long-term consequences of KDB goniotomy are not fully studied and remain the subject of ongoing scientific research.

Costs are also part of the difference between the two devices. The KDB is typically 2–3 times less expensive per procedure than iStent. However, in Poland, this type of surgery is reimbursed by the National Health Fund.

The Kahook Dual Blade is a single-use, disposable device. It is pre-packaged, sterile, and designed for one-time use per eye/patient. Reuse is not approved or recommended due to contamination risk and reduced performance. Moreover, the blade can become dull after a single pass, making reuse clinically unsafe.

According to the available literature, there are no specific recommendations regarding the use of either glaucoma surgery method at a particular age. Moreover, no strict lower or upper age limit is defined in official directives. iStent implantation and KDB goniotomy were primarily evaluated in adult populations (typically aged 45–85) in clinical trials. It has not been formally studied or approved for pediatric patients or adolescents. Some surgeons may consider off-label use in younger adult patients, but data on safety and efficacy below age 40 is limited.

The combination of glaucoma surgery with phacoemulsification also enhances the hypotensive effect, as demonstrated by Barkander et al. and Armstrong et al. [14,20]. Chen PP et al. [21] reported that phacoemulsification alone reduced IOP by up to 30% and glaucoma medications by 58%. In the Gaskin et al. study [11], it was shown that after 4 weeks, the IOP in the phaco-iStent group was lower, but after 24 months, the results were comparable to the phacoemulsification-alone group. This fact supports the efficacy of cataract surgery as a standalone procedure for reducing intraocular pressure. Armstrong et al. analyzed 32 studies involving patients with POAG undergoing phacoemulsification surgery and observed reductions in IOP and glaucoma medications. These outcomes persisted for at least 36 months, although a gradual decline in the initial effect was noted after 1 year [22].

Both MIGS methods described above have a high safety profile, with a low incidence of intraoperative and postoperative adverse events. Backward blood flow into the anterior chamber during the operation is common in MIGS procedures [22]. Blood reflux occurs when Schlemm’s canal is exposed during intraoperative hypotony and should be regarded as an expected occurrence. In one KDB goniotomy study [6], the incidence of hyphema was higher (62%) than in others (<15%). The remaining complications occurred less frequently: IOP spikes (≤14%), PCO (≤3.8%), and others. In one of the studies [11], two intraoperative complications were reported with iStent implantation. The adverse events observed in the compared studies mostly resolved spontaneously without medical intervention. Table 4 summarizes data on adverse events. Despite the complications observed, reductions in intraocular pressure and the use of antiglaucoma eye drops were achieved using both methods.

Despite the utility of our analysis, it is subject to some limitations, such as heterogeneity among the included studies. The variability was influenced by multiple factors, including differences in the severity of glaucoma, number of eyes analyzed, initial intraocular pressure, medications used, levels of surgical expertise, study population characteristics, and demographic profiles. Moreover, owing to practical constraints and safety concerns, most studies were performed without a washout period before surgery; thus, it is difficult to determine the untreated IOP in these patients. Another limitation is that the above-mentioned studies used different outcomes to report their results. A comparison is enabled only by studies reporting the same outcomes (the same criteria of success rate) in a similar fashion. The development of unified methodologies would enhance the interpretation and transparency of study results and facilitate comparisons among reported surgery methods.

Even with these variations, all studies demonstrated reductions in IOP and medication use, underscoring the efficacy of the antiglaucoma methods reviewed.

To summarize, this review article has shown that iStent implantation and goniotomy using KDB, combined with phacoemulsification, effectively lower intraocular pressure and reduce medication use in patients with open-angle glaucoma. These techniques highlight the advantages of the procedure across different device generations, varying numbers of stents, and follow-up periods extending up to 36 months. These findings suggest that either technique may have significant clinical relevance (Table 6).

## 5. Conclusions

Our evaluation reveals that both iStent implantation and KDB goniotomy are valuable techniques for lowering IOP and reducing the number of antiglaucoma drops in patients with OAG. Moreover, both methods have comparable safety profiles and a small number of complications, most of which resolved spontaneously without medical intervention. Most confirmed that MIGS decreased the medication burden, which is otherwise linked with ocular surface issues.

## Figures and Tables

**Table 1:** Risk of bias and overall quality for individual studies.

Bias Category/Study	Barkander et al., 2023 [6]	Dorairaj et al., 2018 [7]	ElMallah et al., 2019 [8]	Lee et al., 2019 [9]	Iwasaki et al., 2019 [10]	Gaskin et al., 2024 [11]		Clement et al., 2022 [12]	Kozera et al., 2021 [13]	Barkander et al., 2023 [14]	Iwasaki et al., 2022 [15]	Dorairaj et al., 2018 [16]
Random sequence generation												
Allocation concealment												
Groups similar at baseline or were differences controlled for?												
Were conditions controlled so effects could be attributed to mobile application?												
Were outcomes prespecified and reported?												
Were participants analyzed based on originally-assigned group across timepoints?												
Were outcome assessors and data analysts masked?												
Were reliable measures of outcomes used consistently across all participants?												
Overall quality (Moderate risk or Low risk of bias)	Moderate	Moderate	Moderate		Moderate	Moderate	Low	Moderate	Low	Moderate	Moderate	Low

**Table 2:** Characteristics of the included studies.

Author/Year	Region	Form of the Procedure of the Studied Group (Control Group)	Size of Studied Group (Control Group)	Follow-Up (Months)	Age of the Patient (Years)
Barkander et al., 2023 [6]	Sweden	KDB Goniotomy + Phacoemulsification	97	24	74.6 ± 5.5
		iStent + Phacoemulsification	56		75.7 ± 7.8
Dorairaj et al., 2018 [7]	Multicenter	KDB Goniotomy + Phacoemulsification	237	6	70.1 ± 8.9
		iStent + Phacoemulsification	198		71.3 ± 8.1
ElMallah et al., 2019 [8]	USA	KDB Goniotomy + Phacoemulsification	190	12	69.6 ± 0.8
		iStent + Phacoemulsification	125		72.4 ± 0.8
Lee et al., 2019 [9]	USA	KDB Goniotomy + Phacoemulsification	44	6	69.1 ± 1.6
		iStent + Phacoemulsification	58		69.5 ± 1.4
Iwasaki et al., 2019 [10]	Japan	KDB Goniotomy + Phacoemulsification	129	12	76.8 ± 7.5
		iStent + Phacoemulsification	44		75.4 ± 7.8
Gaskin et al., 2024 [11]	Australia	iStent + Phacoemulsification	56	24	73.3 ± 7.5
		(Phacoemulsification Solo)	(48)		
Clement et al., 2022 [12]	Multicenter	iStent + Phacoemulsification	273	36	72.4 ± 8.3
Kozera et al., 2021 [13]	Poland	iStent + Phacoemulsification	44	24	70.1 ± 8.5
		(Phacoemulsification Solo)	(36)		
Barkander et al., 2023 [14]	Sweden	KDB Goniotomy + Phacoemulsification	51	24	7.6 ± 6.0
		KDB Goniotomy Solo	39		
Iwasaki et al., 2022 [15]	Japan	KDB Goniotomy + Phacoemulsification	148	31.3 ± 14.8	76.9 ± 7.2
Dorairaj et al., 2018 [16]	North America	KDB Goniotomy + Phacoemulsification	52	12	≥18

**Table 3:** Study characteristics: number of eyes, pre- and postoperative BCVA, IOP, and number of glaucoma drugs applied before and after iStent micro-bypass implantation.

Author/ Year	Number of Eyes: Studied Group (Control Group)	Baseline IOP (mmHg)	Endpoint IOP (mmHg)	Number of Baseline Medication	Number of Endpoint Medications	Mean Preoperative BCVA	Endpoint BCVA
Barkander et al., 2023 [6]	56	20.3 ± 6.1	14.2 ± 4.1	3.0 ± 0.9	2.6 ± 1.1	LOD	LOD
Dorairaj et al., 2018 [7]	198	16.7 ± 4.4	13.9 ± 2.7	1.9 ± 0.9	1.0 ± 1.0	0.4 ± 0.3	0.1 ± 0.2
ElMallah et al., 2019 [8]	125	16.7 ± 0.3	14.4 ± 0.3	1.51 ± 0.06	0.56 ± 0.07	0.34 ± 0.03	0.1 ± 0.01
Lee et al., 2019 [9]	40	16.7 ± 0.4	14.2 ± 0.4	1.4 ± 0.14	1.4 ± 0.14	LOD	LOD
Iwasaki et al., 2019 [10]	44	17.8 ± 2.9	14.3 ± 2.3	2.2 ± 1.1	0.9 ± 1.4	LOD	LOD
Gaskin et al., 2024 [11]	56 (48)	17.7 ± 4.0	15.2 ± 5.8	1.69 ± 1.05	0.7 ± 0.9	0.2 ± 0.2	0.06 ± 0.35
Clement et al., 2022 [12]	273	16.4 ± 4.6	13.9 ± 3.5	1.51 ± 1.17	0.48 ± 0.89	LOD	LOD
Kozera et al., 2021 [13]	44 (36)	22.04 ± 1.64	15.57 ± 2.13	1.32 ± 0.55	0.32 ± 0.55	0.56 ± 0.23	0.95 ± 0.12

BCVA—best-corrected visual acuity; IOP—intraocular pressure; LOD—lack of data.

**Table 4:** Study characteristics: iStent generation and the number of implants inserted.

Author/Year	iStent Generation	Number of Implants	Number of Eyes
Barkander et al., 2023 [6]	second	2	56
Dorairaj et al., 2018 [7]	first	1	198
ElMallah et al., 2019 [8]	first	1	125
Lee et al., 2019 [9]	first	1	40
Iwasaki et al., 2019 [10]	first	1	44
Gaskin et al., 2024 [11]	second	3	1
		2	54
		1	1
Clement et al., 2022 [12]	second	2	271
		1	2
Kozera et al., 2021 [13]	first	1	44

**Table 5:** Study characteristics: follow-up, number of eyes, pre- and postoperative BCVA, IOP, and number of glaucoma drugs applied before and after KDB goniotomy.

Author/ Year	Number of Eyes: Studied Group (Control Group)	Baseline IOP (mmHg)	Endpoint IOP (mmHg)	Number of Baseline Medications	Number of Endpoint Medications	Mean Preoperative BCVA	Endpoint BCVA
Barkander et al., 2023 [6]	97	20.1 ± 6.1	14.7 ± 3.6	2.3 ± 1.0	1.5 ± 1.3	LOD	LOD
Dorairaj et al., 2018 [7]	237	17.9 ± 4.4	13.6 ± 2.7	1.7 ± 0.9	0.6 ± 1.0	0.4 ± 0.3	0.1 ± 0.2
ElMallah et al., 2019 [8]	190	18.2 ± 0.3	13.2 ± 0.1	1.45 ± 0.05	0.43 ± 0.05	0.34 ± 0.02	0.1 ± 0.01
Lee et al., 2019 [9]	34	17.2 ± 0.7	14.8 ± 0.6	1.9 ± 0.17	1.0 ± 0.17	LOD	LOD
Iwasaki et al., 2019 [10]	44	19.8 ± 7.3	13.0 ± 3.1	2.5 ± 1.4	1.6 ± 1.6	LOD	LOD
Barkander et al., 2023 [14]	51 (39)	22.3 ± 5.8	13.9 ± 3.0	3.3 ± 0.5	2.3 ± 1.1	0.25 ± 0.30	LOD
Iwasaki et al., 2022 [15]	148	19.5 ± 6.9	11.9 ± 2.7	2.4 ± 1.4	1.6 ± 1.4	0.53 ± 0.46	0.35 ± 0.67
Dorairaj et al., 2018 [16]	52	16.8 ± 0.6	12.4 ± 0.3	1.6 ± 0.2	0.8 ± 0.1	0.439 ± 0.041	0.137 ± 0.016

BCVA—best-corrected visual acuity; IOP—intraocular pressure; LOD—lack of data.

**Table 6:** The most common postoperative adverse events after surgery.

Author/ Adverse Event	Barkander et al. n (%) [6]		Dorairaj et al. n (%) [7]		ElMallah et al. n (%) [8]		Lee et al. n (%) [9]		Iwasaki et al. n (%) [10]		Gaskin et al. n (%) [11]	Clement et al. n (%) [12]	Kozera et al. n (%) [13]	Barkander et al. n (%) [14]	Iwasaki et al. n (%) [15]	Dorairaj et al. n (%) [16]
Type of MIGS	iStent	KDB	iStent	KDB	iStent	KDB	iStent	KDB	iStent	KDB	iStent	iStent	iStent	KDB	KDB	KDB
Hyphema	7 (14%)	60 (62%)	1 (0.5%)	9 (3.8%)	NR	NR	NR	2 (4.5%)	1 (2.3%)	21 (16.3%)	NR	NR	5 (11.4%)	2 (4%)	21 (14.2%)	NR
IOP spikes	2 (5%)	13 (14%)	25 (12.6%)	15 (6.3%)	1 (0.8%)	2 (1%)	1 (1.7%)	8 (18.2%)	3 (6.8%)	18 (14%)	NR	NR	1 (1.25%)	4 (8%)	19 (12.8%)	2 (3.8%)
Stent obstruction	NR	NA	NR	NA	NR	NA	NR	NA	NR	NA	NR	2 (<1%)	NR	NA	NA	NA
Stent malposition	NR	NA	NR	NA	NR	NA	NR	NA	1 (2.3%)	NA	2 (3.57%)	NR	NR	NA	NA	NA
Macular edema	NR	1 (1.03%)	NR	NR	NR	NR	1 (1.7%)	2 (4.5%)	NR	NR	NR	NR	NR	1 (2%)	NR	1 (1.9%)
Corneal edema	NR	NR	3 (1.5%)	5 (2.1%)	1 (0.8%)	2 (1%)	NR	NR	NR	NR	NR	NR	1 (1.25%)	NR	NR	NR
Inflammation	3 (5%)	3 (5%)	4 (2%)	1 (0.4%)	3 (2.4%)	2 (1%)	NR	NR	NR	NR	NR	NR	NR	1 (2%)	NR	NR
Additional procedures	7 (12.5%)	4 (4.12%)	NR	NR	NR	NR	NR	NR	NR	3 (2.3%)	4 (7.2%)	23 (8.42%)	NR	6 (12%)	6 (4.1%)	NR
Pain/ irritation	NR	NR	NR	NR	NR	NR	NR	NR	NR	NR	NR	NR	NR	NR	NR	4 (7.7%)
PCO	NR	3 (3.09%)	5 (2.5%)	1 (0.4%)	1 (0.8%)	3 (1.6%)	NR	NR	NR	NR	NR	NR	4 (9.1%)	NR	NR	2 (3.8%)
Posterior vitreous detachment	NR	NR	2 (1%)	2 (0.8%)	NR	NR	NR	NR	NR	NR	NR	NR	NR	NR	NR	NR
Rebound iritis	NR	NR	2 (1%)	2 (0.8%)	NR	NR	NR	NR	NR	NR	NR	NR	NR	NR	NR	NR

NR—not reported; NA—not applicable; PCO—posterior capsular opacification.

## Data Availability

All data are available to the public.

## Abbreviations

The following abbreviations are used in this manuscript:

IOP	intraocular pressure
KDB	Kahook Dual Blade
MIGS	microinvasive glaucoma surgery
OAG	open-angle glaucoma
PCO	posterior capsular opacification
PEXG	pseudo-exfoliation glaucoma
PG	pigmentary glaucoma
POAG	primary open-angle glaucoma
SC	Schlemm’s canal
TM	trabecular meshwork
